# Higher Soong grade predicts flexor tendon issues after volar plating of distal radius fractures – a retrospective cohort study

**DOI:** 10.1186/s12891-023-06313-0

**Published:** 2023-04-10

**Authors:** Henri Vasara, Petra Tarkiainen, Antti Stenroos, Jussi Kosola, Turkka Anttila, Anni Aavikko, Panu H Nordback, Samuli Aspinen

**Affiliations:** 1 Department of Hand Surgery, Helsinki University Hospital and University of Helsinki, Helsinki, Finland; 2 Department of Orthopedics and Traumatology, Helsinki University Hospital and University of Helsinki, Helsinki, Finland; 3 Department of Orthopedics and Traumatology, Kanta-Häme Central Hospital and University of Helsinki, Hämeenlinna, Finland; 4 Department of Orthopedics and Traumatology, Päijät-Häme Central Hospital and University of Helsinki, Lahti, Finland

**Keywords:** Distal Radius fracture, Volar locking plate, Soong classification, Flexor pollicis longus rupture, Flexor rupture, Plate removal, Flexor tendon issues

## Abstract

**Background and purpose:**

Soong classification is used to estimate volar locking plate prominence and evaluate the risk for flexor tendon ruptures after surgical treatment of distal radius fractures (DRFs). However, the scientific community has questioned the Soong classification due to lacking evidence. Therefore, this study aimed to evaluate the accuracy of Soong grading as a predictor for flexor tendon issues and plate removal.

**Patients and methods:**

We performed a retrospective single-center review of adult distal radius fracture patients treated with a volar locking plate between 2009 and 2019. In total, 2779 patients were included in the study. The primary outcome was a flexor tendon issue (flexor tendon rupture, tendinitis, or flexor irritation), whereas plate removal was a secondary outcome. Using Soong grade 0 as a reference, we used univariable and multivariable logistic regression to calculate odds ratios (OR) with 95% confidence intervals (CI) for flexor tendon issues and plate removal.

**Results:**

In total, 756 (27%) patients were graded as Soong 0, 1679 (60%) Soong 1, and 344 (12%) Soong 2, respectively. There were 32 (1.2%) patients with flexor tendon issues, of which 4 were flexor tendon ruptures, 8 tendinitises, and 20 flexor irritations. The adjusted OR for flexor tendon issues was 4.4 (CI 1.1–39.7) for Soong grade 1 and 9.7 (CI 2.2–91.1) for Soong grade 2. The plate was removed from 167 (6.0%) patients. Soong grade 1 had a univariable OR of 1.8 (CI 1.2–2.8) for plate removal, and Soong grade 2 had an OR of 3.5. (CI 2.1–5.8), respectively.

**Conclusion:**

Flexor tendon ruptures are rare complications after the volar plating of DRFs. A higher Soong grade is a risk factor for flexor tendon issues and plate removal.

**Trial registration:**

The trial was retrospectively registered.

## Introduction

Volar locking plates (VLPs) have become a prevalent method of fixation for the surgical treatment of adult distal radius fractures (DRFs) [1, 2]. Operative treatment with volar locking plates allows a desirable fracture reduction and accelerates the recovery of the wrist function compared to other DRF treatment options, although long-term patient reported outcomes do not differ between fixation methods [3–5]. Nowadays, 15–25% of adult DRFs are managed with internal plate fixation [1, 6]. However, with the expanding use of VLPs, addressing complications has increasingly arisen in the discussion.

A well-recognized complication of VLPs is flexor tendon ruptures, especially flexor pollicis longus (FPL) ruptures. These complications are considered to arise from plate design, plate position, poor reduction, and fixation failure. Initially, the incidence of flexor ruptures was reported to be as high as 1.9% [7], but has since decreased, presumably due to improved surgical technique, plate design, and increasing prevalence of the procedure. In a recent systematic review, the reported flexor tendon rupture incidence was 0.3% [8].

Soong et al. introduced a classification in 2011 that has since been used to estimate volar plate prominence [9] Accordingly, volar plates positioned more prominently and closer to the volar rim have a greater risk of flexor tendon ruptures. However, although Soong classification is commonly used, the correlation between different Soong grades and flexor tendon issues has not been established [10–13].

This study aimed to evaluate the accuracy of Soong grading as a predictor for flexor tendon issues (ruptures, tendinitises, and irritations). We hypothesized there is no clinically significant difference in the incidence of flexor tendon issues between Soong grade 0 and 1 plates. Furthermore, we hypothesized that prominent plates should not be routinely removed.

## Patients and methods

### Study design

We performed a retrospective single-center cohort study between January 2009 and December 2019. We identified all distal radius fractures treated with a volar locking plate in Helsinki University Hospital, a level I trauma center responsible for surgical fracture treatment in Helsinki metropolitan area. The patients were followed up retrospectively from the electronic patient information system. The study’s primary outcome was a flexor tendon issue (flexor tendon rupture, tendinitis, or irritation), whereas plate removal was a secondary outcome.

We identified all operatively treated DRF patients with ICD-10 code S52.5 (fracture of the distal radius), and Nordic Medico-Statistical Committee (NOMESCO) procedure codes NCJ62, NCJ64, NDJ62, or NDJ64 (forearm or wrist fracture surgery with a plate, screws, or K-wires). The inclusion criteria for the study were skeletally mature patients (aged > 18) with a VLP as the primary fixation method. Patients with additional K-wires were included in the study if the K-wires were planned to be removed. Patients with dorsal plates, additional screws, fragment-specific plates, or external fixators were excluded from the study. Lack of appropriate radiographs, prior flexor tendon injury, and insufficient clinical follow-up (< 28 days) were also considerations for exclusion. In total, 2779 patients were included in the study.

### Patient demographics and surgical details

The authors examined perioperative patient records and collected the patient demographics (Table 1).

All operations were performed by hand surgery, or orthopedic specialists, or senior residents (> 3 years of surgical experience) following AO principles [14]. The operations were done under fluoroscopy, a tourniquet was used routinely, and the modified Henry method was the standard approach. The Pronator quadratus muscle was sutured according to the surgeon’s preference, and the intel was recorded if available.

The operating surgeon chose the volar plate based on fracture morphology and personal preference. The following volar plates were used for the fracture reduction: Acu-Loc 2 (Acumed LLC, Hillsboro, Oregon) in 1602 (58%), DVR plate (Zimmer Biomet, Warsaw, Indiana) in 389 (14%), Variable Angle LCP Two-column VDR Plate 2.4 (DePuy Synthes, Oberdorf, Switzerland) in 199 (7%), LCP Volar Column Distal Radius Plate 2.4 (DePuy Synthes, Oberdorf, Switzerland) in 231 (8%), Acu-Loc 1 (Acumed LCC, Hillsboro, Oregon) in 199 (7%), and various unknown other plates in 83 (3%) cases, respectively.

### Fracture classification

We analyzed all fracture-related radiographs from the hospital Picture Archiving and Communication System. Standard plain radiographs with postero-anterior and lateral projections were available for all patients. In addition, computed tomography scans were observed when available. The radiograph analysis was done by a single author (HV, PT, TA, PN, AA, AS, or SA). Complex cases of fracture morphology, Soong classification, or fixation evaluation were examined together by all authors, and a conclusion was made in unison. In addition, all radiographs with flexor tendon issues were reviewed and graded together with all authors.

**Fig. 1:**
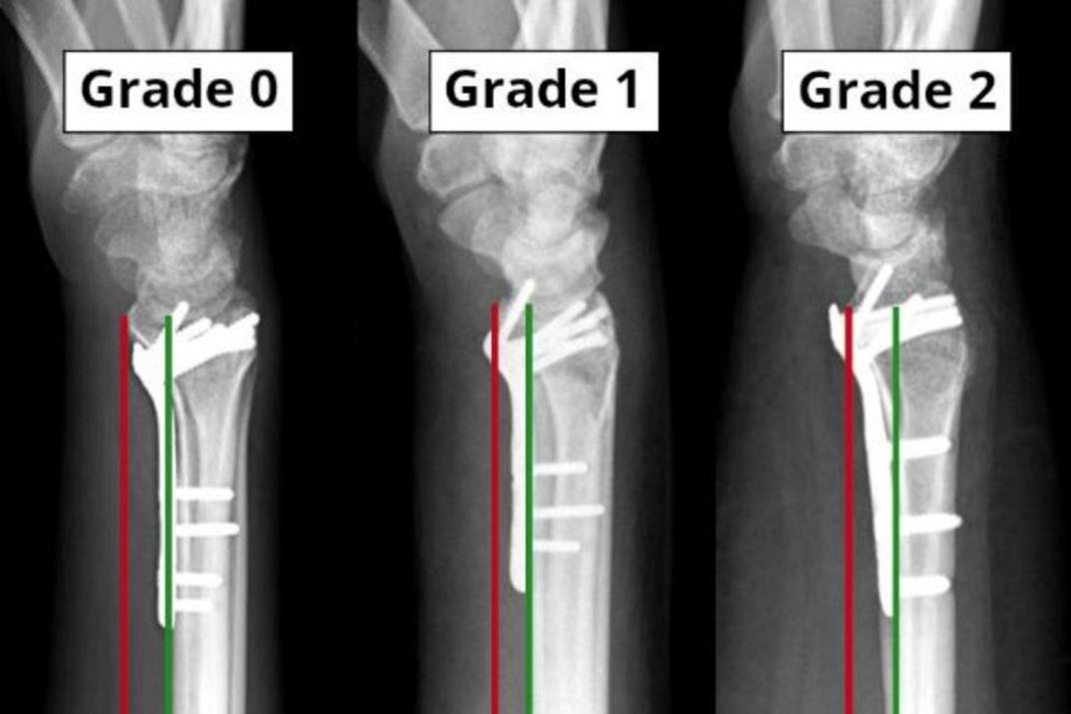
The Soong classification The red line marks the critical line drawn parallel to the volar cortex of the radius diaphysis (green line)

We gave each patient a Soong grade according to the system proposed by Soong et al. [9]. A lateral projection with the thinnest plate profile was chosen from the postoperative radiographs. An imaginary “critical line” parallel to the volar cortex of the radius was drawn via the volar rim of the radius. Accordingly, if the plate was proximal to the volar rim and did not exceed the critical line, a Soong grade 0 was given. Plates proximal to the volar rim, exceeding the critical line, were graded as Soong 1, and plates distal to the volar rim were graded as Soong 2 (Fig. 1).

The fracture morphology was classified according to the AO fracture classification system from the preoperative radiographs [15]. Furthermore, we evaluated the fracture fixation based on the postoperative radiographs. The fracture fixation was considered acceptable if radiocarpal joint surface diastasis or step-off was less than 1 mm, the radial inclination was between 10–30°, radio-ulnar shortening was less than 2 mm, dorsal tilt was less than 5°, and ad latus shift affecting the distal radioulnar joint (DRUJ) was less than 1 mm.

### Follow-up

All patients had at least one follow-up visit at the outpatient clinic 28–42 days after the operation. Additional outpatient clinic visits were scheduled when considered necessary by the treating physician. We followed up the patients from the electronic patient information system for a minimum of one year. We examined all fracture-related visits in the outpatient clinic and emergency department in case of flexor tendon-related issues or plate removal. The median time from the surgery to the last follow-up visit was 43 days (IQR 36–99), and the median time between the injury and the date we accessed the data was 5.8 years (IQR 3.1–8.5 years).

### Outcome

We analyzed each case of flexor tendon rupture, tendinitis, flexor irritation, and plate removal in detail. Flexor tendon rupture was classified as a complete absence of the corresponding finger movement, examined by a physician. The tendinitises were confirmed with imaging modalities or clinical signs during a secondary operation. Flexor irritation was classified as physician-confirmed tenderness, swelling, or pain on the volar distal radius, worsened by finger or wrist movement.

### Statistical analysis

Categorical variables were examined using cross-tabulations and presented as counts and percentages. The normality of continuous variables was tested visually using histograms and Q-Q plots. As all continuous variables were non-normal, we presented them as medians (interquartile range). We used the Wilson score interval to calculate the 95% confidence interval (CI) to estimate the incidence proportions of flexor tendon issues and plate removal.

**Fig. 2:**
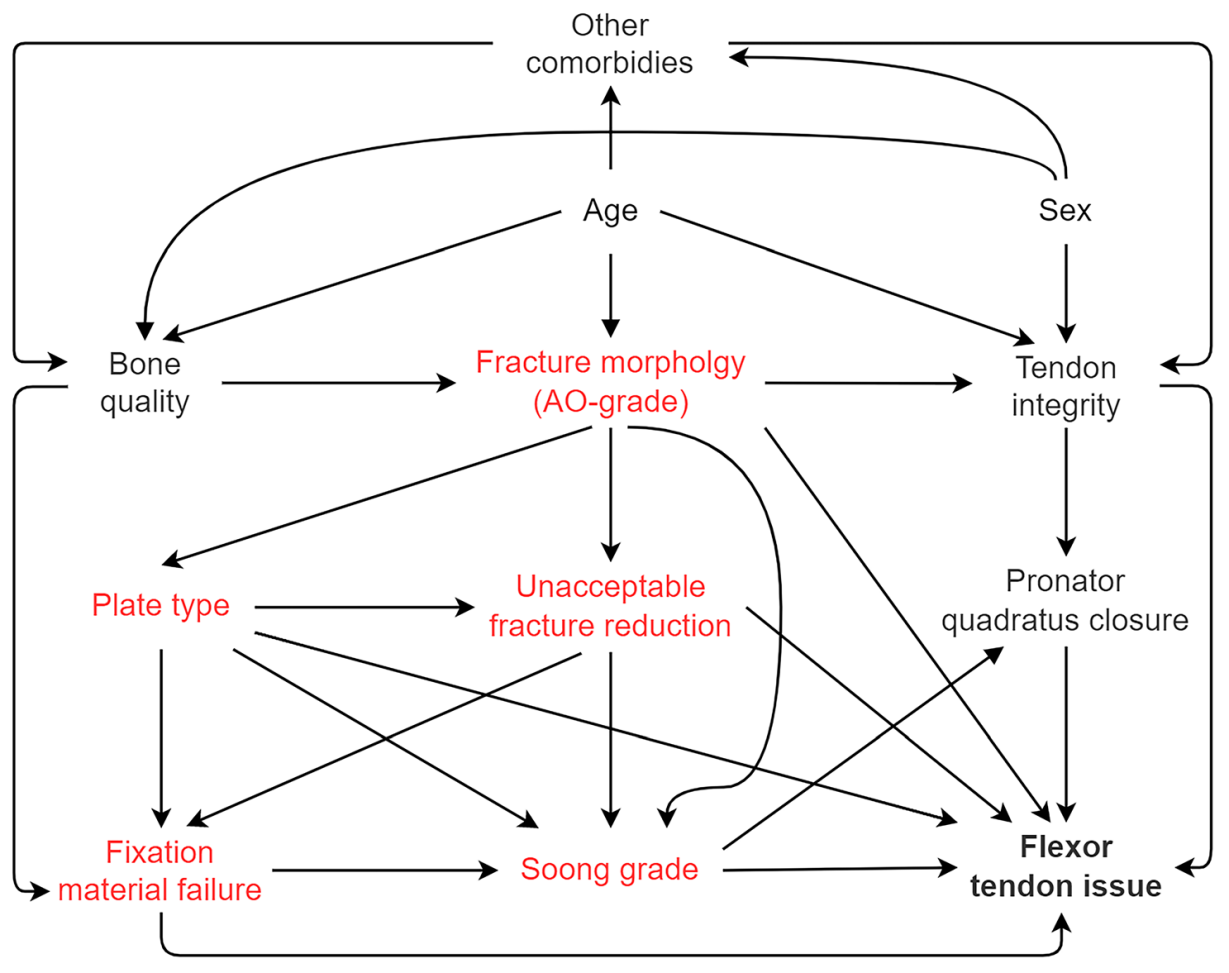
The directed acyclic graph (DAG) we used to determine the multivariable model’s confounding factors Variables in red were included in the model

First, we used univariable binary logistic regression to evaluate the Soong grade as a risk factor for flexor tendon issues and plate removal. Using Soong grade 0 as a reference value, odds ratios (OR) with 95% CI were calculated for Soong 1 and Soong 2. Second, we implemented a multivariable analysis for flexor tendon issues. We constructed a Directed Acyclic Graph (DAG) to avoid bias in detecting the confounding factors included in the multivariable analysis (Fig. 2) [16]. When conducting the adjustments, we noticed some subgroups contained zero events, thus making the initial binary logistic regression method inoperable. According to a biostatistician’s consultation, we chose Firth’s Logistic Regression for rare events to conclude the multivariable analysis. We used the statistical program SPSS 28.0 (IBM corp. released on the 10th of November 2021) with STATS_FIRTHLOG-extension 1.0.6 (IBM corp. released on the 12th of December 2019) for the analyses.

## Results

In total, there were 756 (27%) patients with Soong 0, 1679 (60%) patients with Soong 1, and 344 (12%) patients with Soong 2 grade plates (total n = 2779). The median age of the patients was 57.2 years (IQR 44.3–65.4), and 69% (n = 1922) of the patients were female. There were 470 (17%) type A fractures, 242 (9%) type B fractures, and 2067 (74%) type C fractures, respectively. The fracture reduction was considered unacceptable in 408 (15%) cases. Precise patient characteristics are presented in Table 1, and a comparison between the plate types is shown in Table 2.

**Table 1:** Patient demographics and perioperative confounding factors. The values are counts (%) if not otherwise specified

	Total (n = 2779)
**Age, years, median (IQR)**	57.2 (44.3–65.4)
**Female sex**	1922 (69.2%)
**Delay to surgery, days, median (IQR)**	8 (5–13)
**Active follow-up, days (IQR)** ^**a**^	43 (36–99)
**AO/OTA classification**	
Type A	470 (16.9%)
Type B	242 (8.7%)
Type C	2067 (74.4%)
**Implant type**	
Acu-Loc 2	1602 (57.6%)
DVR	389 (14.0%)
Synthes 2,4 collum	275 (9.9%)
Acu-Loc 1	231 (8.3%)
Synthes variable angle LCP	199 (7.1%)
Other	83 (3.0%)
**Pronator quadratus left unclosed** ^**b**^	100 (3.8%)
**Acceptable fracture reduction** ^**c**^	2371 (85.3%)
**Fixation failure**	37 (1.3%)

a. Time between the surgery and last fracture-related hospital visit

b. Missing values n = 132

c. Dorsal tilt < 15°, inclination 10–30°, diastasis or step-off < 1 mm, and ad latus in DRUJ < 1 mm

**Table 2:** The relationship between plate type and different confounding factors. The values are counts (%), and the percentages are compared against the volar plate (column) in question

	Acu-Loc 2 (n = 1602)		DVR (n = 389)		Synthes 2,4 mm column (n = 275)		Acu-Loc 1 (n = 231)		Synthes variable angle LCP (n = 199)		Other plates (n = 83)	
AO/OTA classification												
Type A	183	(11.4%)	128	(32.9%)	58	(21.1%)	23	(10.0%)	39	(19.6%)	34	(41.0%)
Type B	125	(7.8%)	41	(10.5%)	32	(11.6%)	17	(7.4%)	25	(12.6%)	3	(3.6%)
Type C	1294	(80.8%)	220	(56.6%)	185	(67.3%)	191	(82.7%)	135	(67.8%)	46	(55.4%)
**Soong grade**												
Soong 0	440	(27.5%)	184	(47.3%)	58	(21.1%)	17	(7.4%)	39	(19.6%)	18	(22%)
Soong 1	993	(62.0%)	194	(49.9%)	177	(64.4%)	145	(62.8%)	128	(64.3%)	42	(50%)
Soong 2	169	(10.5%)	11	(2.8%)	40	(14.5%)	69	(29.9%)	32	(16.1%)	23	(27%)
**Acceptable fracture reduction after operation** ^**a**^	1396	(87.1%)	346	(88.9%)	228	(82.9%)	175	(75.8%)	164	(82.4%)	62	(74.7%)
**Fixation failure**	13	(0.8%)	2	(0.5%)	8	(2.9%)	7	(3.0%)	5	(2.5%)	2	(2.4%)
**Flexor tendon issues**	14	(0.9%)	0		2	(0.7%)	10	(4.3%)	6	(3.0%)	0	
Irritation	11	(0.7%)	0		0		6	(2.6%)	3	(1.5%)	0	
Tendinitis	2	(0.1%)	0		2	(0.7%)	2	(0.9%)	2	(1.0%)	0	
Rupture	1	(0.1%)	0		0		2	(0.9%)	1	(0.5%)	0	
**Plate removal**	96	(6.0%)	14	(3.6%)	16	(5.8%)	18	(7.8%)	18	(9.0%)	5	(6.0%)

a. Dorsal tilt < 15°, inclination 10–30°, diastasis or step-off > 1mm, and ad latus in DRUJ < 1mm

### Flexor tendon issues

**Fig. 3:**
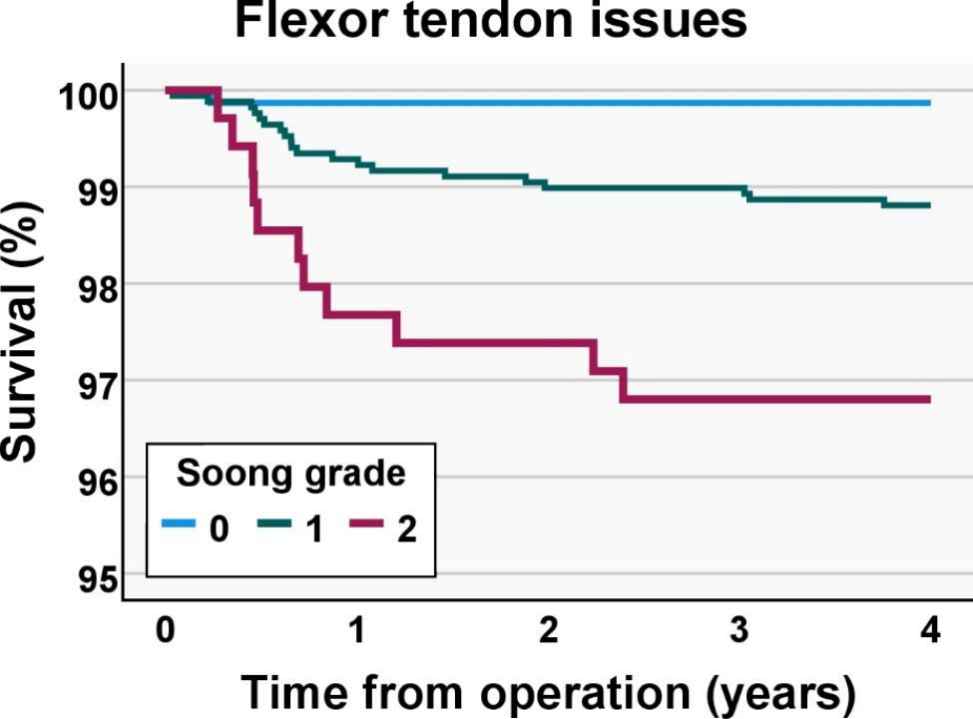
Kaplan-Meier survival plot for flexor tendon-related issues

**Fig. 4:**
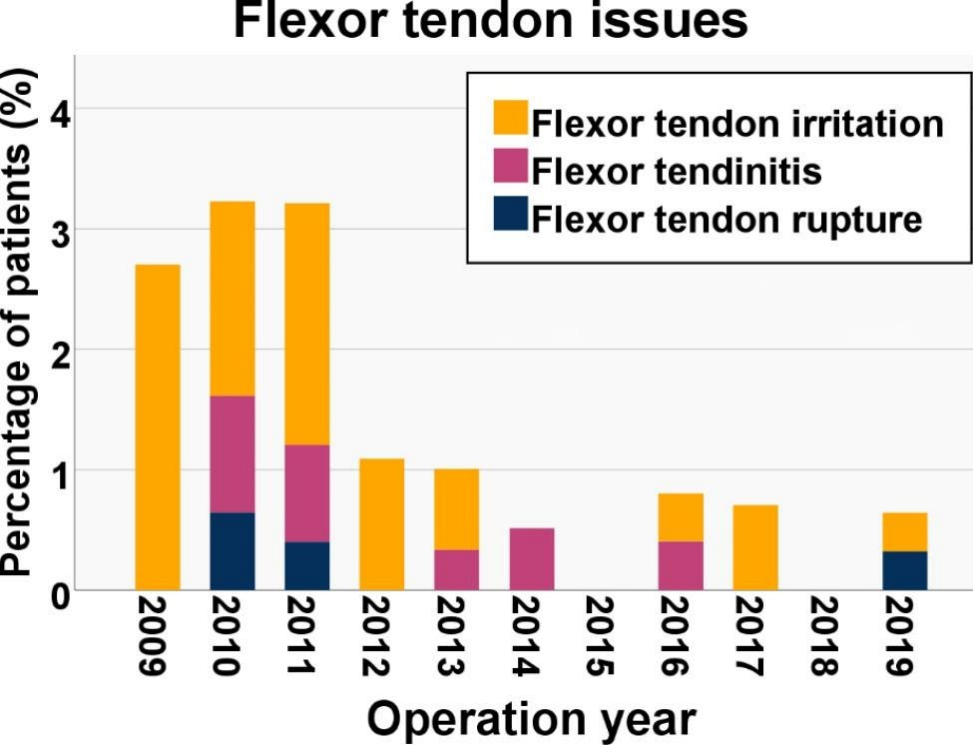
Yearly incidences (%) of patients with flexor tendon issues

There were 32 patients (incidence 1.2%, CI 0.8–1.6%) with flexor tendon issues (Table 3). These included 4 (0.1%, CI 0.0-0.3%) patients with flexor tendon ruptures, 8 (0.3%, CI 0,1 − 0,5%) with tendinitis, and 20 (0.7%, 0,5 − 1,1%) with a physician-diagnosed flexor tendon irritation. The flexor tendon issues were emphasized in the first year post-operatively, as 21 (66%) were detected during that period (Fig. 3). The yearly incidence of flexor tendon issues was considerably higher for patients who were operated on between the years 2009–2011 (incidence 2.0 − 3.3%) than after 2012 (incidence 0.0 − 1.1%) (Fig. 4).

Overall, there were 5 flexor tendon ruptures in 4 patients. The ruptures were diagnosed a median of 494 days after the operation (range 81-1115 days). In two cases, the affected tendon was the FPL, in one case, the flexor digitorum profundus 2 (FDP2), and in one case, both FPL and FDP2. Acu-Loc 1 plate was used in two cases, and Acu-Loc 2 and Synthes variable angle plates were both used in a single patient with flexor tendon ruptures (Table 2).

**Table 3:** Flexor tendon-related issues and plate removal by Soong grade

	Soong 0 (n = 756)	Soong 1 (n = 1679)		Soong 2 (n = 344)		Total (n = 2779)
	**n (%)**	**n (%)**	**OR (95% CI)**	**n (%)**	**OR (95% CI)**	**n (%)**
**Flexor tendon-related issues**	1 (0.1%)	19 (1.1%)	**9.1 (1.2–68)**	12 (3.6%)	**24.9 (3.2–194)**	32 (1.2%)
Flexor tendon rupture	0	2 (0.1%)	-	2 (0.6%)	-	4 (0.1%)
Tendinitis	0	2 (0.1%)	-	6 (1.7%)	-	8 (0.3%)
Flexor irritation	1 (0.1%)	15 (0.9%)	-	4 (1.2%)	-	20 (0.7%)
**Plate removal**	26 (3.4%)	103 (6.1%)	**1.8 (1.2–2.8)**	38 (11.0%)	**3.5 (2.1–5.8)**	167 (6.0%)

Soong grade 0 is used as a reference for odds ratios (OR). The percentages are compared against the Soong grade (column) in question

### Risk for flexor tendon issues

The incidence of flexor tendon issues was significantly greater in Soong 1 (1.1%, CI 0.7–1.7%) and Soong 2 (3.6%, CI 1.9–5.8%) grade plates when compared with Soong 0 (0,1%, CI 0.0-0.6%). Using Soong 0 as a reference, the univariable odds ratio for flexor tendon issues was 9.1 (CI 1.2–68) for Soong 1 and 24.9 (CI 3.2–194) for Soong 2, respectively (Table 3). When adjusted with fracture morphology (AO grade), plate type, acceptable primary fixation, and fixation failure, Soong 1 had an adjusted OR of 4.4 (CI 1.1–39.7, p = 0.03) and Soong 2 an adjusted OR of 9.7 (CI 2.2–91.1, p = 0.001).

### Plate removal

**Fig. 5:**
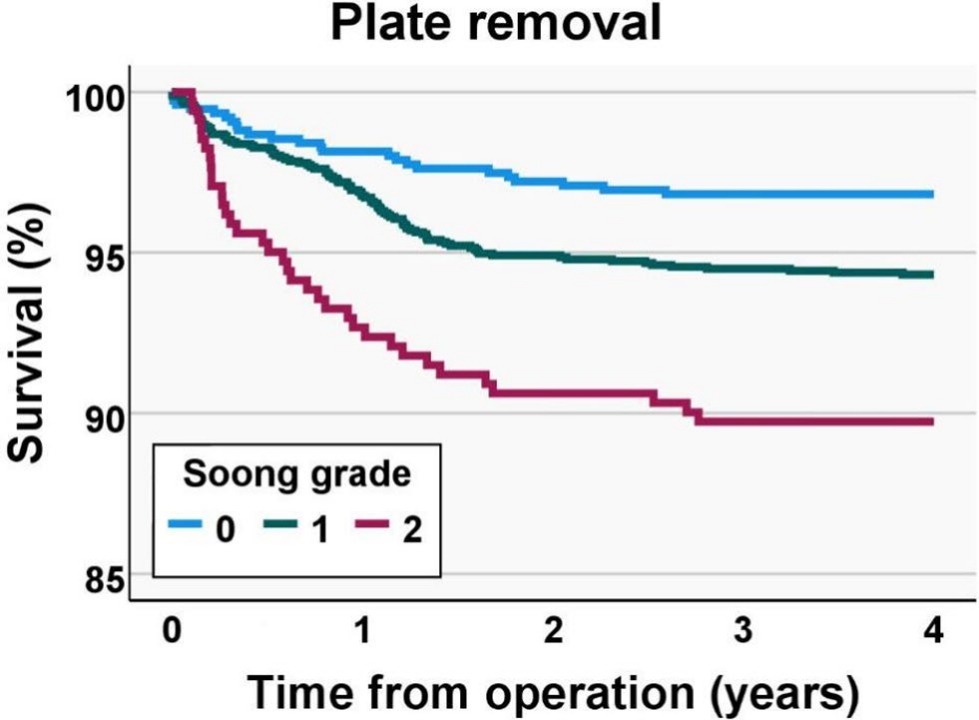
Kaplan-Meier survival plot for plate removal

**Fig. 6:**
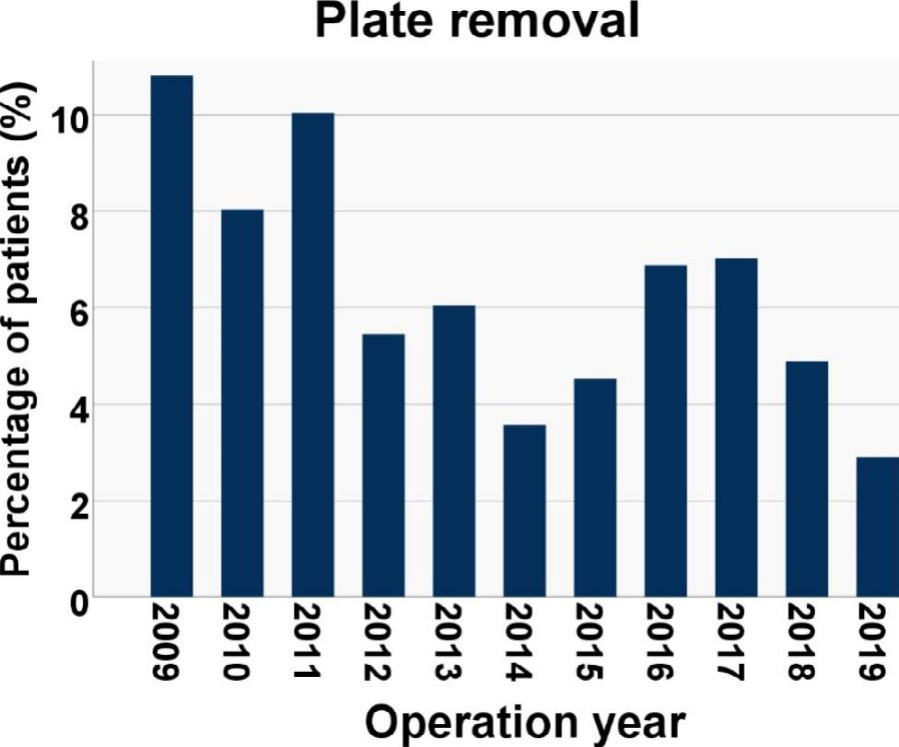
Yearly incidences (%) of patients with flexor tendon issues

The volar locking plate was removed from 167 (6%, CI 5–7%) patients. The incidence of plate removal was significantly greater in Soong 1 (6.1%, CI 5.1–7.4%) and Soong 2 (11.0%, CI 8.1–14.7%) grade plates when compared with Soong 0 (3.4%, CI 2.3–4.9%). In the univariable analysis, the odds ratio for plate removal was 1.8 (1.2–2.8) for Soong 1 and 3.5 (2.1–5.8) for Soong 2 grade plates (Table 3). Plate removal was emphasized in the first two years post-operatively (Fig. 5). The rate for plate removal had a decreasing tendency during the study period, as 8.2–10.5% of patients operated on in 2009–2011 had their plates removed, whereas, during 2012–2019, the corresponding rate was 3.1–7.1% (Fig. 6).

## Discussion

The present study demonstrated that flexor tendon issues (1.2%) and flexor ruptures (0.1%) are rare after distal radius fracture surgery with a volar locking plate. Contrary to our hypothesis, the Soong grade was a solid predictor for flexor tendon issues and plate removal, as a higher Soong grade was directly proportional to higher flexor tendon issues and plate removal incidences. Overall, our study presents important evidence that more proximal plate positioning is desirable if it can be achieved without affecting the reduction and stability. However, the classification’s clinical relevance must be evaluated accounting also the minimal incidence of the outcome.

The incidences of flexor tendon ruptures and tendinitis were 0.3% and 0.7% in a systematic review done in 2021, respectively [8]. As a comparison, a 2017 systematic review reported incidences of 0.7% for flexor tendon ruptures and 2.5% for tendinitis [17]. Furthermore, our large cohort revealed that only 0.1% of patients sustained a flexor tendon rupture, and 0.3% had tendinitis. Accordingly, flexor tendon ruptures are nowadays a rare complication, and the trend seems to be decreasing.

Several publications have examined the impact of Soong grade as a predictor of complications after fixation of distal radius fractures with a VLP [10, 12, 13, 18–22] However, only a few publications have a sufficient number of patients for adequate statistical analysis [10, 21] In addition, although the original purpose of the Soong classification was to predict FPL ruptures [9], the classification has been widely adapted to predict other factors, such as plate removal and reoperations [12, 21].

The association between flexor tendon ruptures and Soong classification has been disputed in the surgeon community managing upper extremity injuries. In their original study, Soong et al. examined 165 patients and found 3 flexor tendon ruptures and 3 plate removals due to flexor irritations. Of these 6 flexor tendon issues, 4 were seen in plates graded as Soong 2. However, in two cases, prominent screws were also present. [9] Previously, DeGeorge et al. and Cook et al. did not find a statistically significant association between Soong classification and flexor tendon issues [13, 19]. In contrast, Kitay et al. and Gören et al. reported a significant correlation [20, 22]. However, Gören et al. examined 113 patients whose plates were removed [22], and Kitay et al. had a case-control study with 8 flexor rupture patients [20]. Thus, the present study is the first to describe a distinct association between Soong classification and flexor tendon issues in a large cohort.

The Soong classification has been associated with plate removal in several studies [21, 23, 24], although there are smaller studies with contradictory findings [11]. Recently, Meyer et al. concluded that Soong 1 had an OR of 16 (CI 5.8–47) and Soong 2 an OR of 44 (CI 14–140) for plate removal when compared to Soong grade 0 plates [21]. Their findings align with ours, although we did not find an as strong effect with OR of 1.8 (CI 1.2–2.8) for Soong 1 and 3,5 (CI 2.1–5.8) for Soong 2. Accordingly, the overall evidence suggests a correlation between Soong grade and plate removal. However, plate removal is not a reliable outcome measurement for Soong classification accuracy as there are numerous contributing factors that cannot be accounted for entirely in the statistical analysis. As the incidence of flexor tendon issues was low, we do not recommend routine plate removal without clinical implications.

There has been discussion regarding the effect of the plate type on flexor tendon issues. In our material, we saw differences in the outcomes between different volar plates. Patients with DVR sustained no flexor tendon issues, whereas Acu-Loc 1 plates had a proportional incidence of 4.3%. However, DVR was used more for A-type fractures, thus getting a higher percentage of patients with Soong grade 0. In addition, In the literature, Meyer et al. reported that DVR had an OR of 0.3 (CI 0.15–0.75) to plate removal compared to Synthes variable angle LCP [21]. Altogether, the fracture morphology often guides the plate selection and positioning, affecting both Soong grade and the risk of getting a tendon issue. Based on our study, we cannot conclude whether specific plates are superior regarding flexor tendon issues or plate removal, although the trend seems to favor plates that can be easily positioned in Soong grade 0.

According to our knowledge, the present study has the most considerable number of patients in the current literature [10, 13, 19, 20], allowing us to perform reliable statistical analysis, even in such a rare complication. The limitations of our study are mainly due to its retrospective nature: First, the available follow-up time varied between the patients, and all flexor tendon issues might not have yet developed. However, most flexor tendon issues developed during the first year, and most patients had a sufficient follow-up. Second, some patients might have their complications treated in other hospitals. Nevertheless, the re-referral rate in our institution is high, and most patients return to us for re-evaluation in case of complications detected in another clinic. Finally, multiple authors participated in the evaluation of the Soong grades, which can affect the grading reliability. However, both Lutzky et al. and Creighton et al. reported high intra- and interobserver reliability in the Soong grading [11, 25]. Furthermore, the Soong grading of the patients with flexor tendon issues was done in unison with all authors, further diminishing the probability of miscategorization in these patients.

## Conclusion

Flexor tendon ruptures are rare complications with an incidence of 0.1% after surgically treated DRFs. Soong classification is a functional tool for plate prominence evaluation, as a higher Soong grade has a higher risk for flexor tendon issues and plate removals. We do not recommend routine plate removal. To avoid flexor tendon issues, surgeons should emphasize plate selection, positioning, and fracture reduction.

## Data Availability

The datasets used and analysed during the current study are available from the corresponding author on reasonable request.

## List of Abbreviations

DRF: Distal Radius Fracture
VLP: Volar Locking Plate
FPL: Flexor Pollicis Longus
FDP: Flexor Digitorum Profundus
CI: Confidence Interval
DAG: Directed Acyclic Graph OR: Odds Ratio
OR: Odds Ratio
